# Of 11 candidate steroids, corticosterone concentration standardized for mass is the most reliable steroid biomarker of nutritional stress across different feather types

**DOI:** 10.1002/ece3.5701

**Published:** 2019-10-02

**Authors:** Alexis Will, Katherine Wynne‐Edwards, Ruokun Zhou, Alexander Kitaysky

**Affiliations:** ^1^ Institute of Arctic Biology University of Alaska Fairbanks Fairbanks Alaska; ^2^ Veterinary Medicine & Hotchkiss Brain Institute University of Calgary Calgary Alberta

**Keywords:** 17‐hydroxyprogesterone, androstenedione, corticosterone, cortisol, cortisone, feather, LC‐MS/MS, testosterone

## Abstract

1. Measuring corticosterone in feathers has become an informative tool in avian ecology, enabling researchers to investigate carry‐over effects and responses to environmental variability. Few studies have, however, explored whether corticosterone is the only hormone expressed in feathers and is the most indicative of environmental stress. Essential questions remain as to how to compare hormone concentrations across different types of feathers and whether preening adds steroids, applied after feather growth.
2. We used liquid chromatography coupled to tandem mass spectrometry to quantify a suite of 11 steroid hormones in back, breast, tail, and primary feathers naturally grown at overlapping time intervals by rhinoceros auklet *Cerorhinca monocerata* captive‐reared fledglings and wild‐caught juveniles. The captive‐reared birds were raised on either a restricted or control diet. Measured steroids included intermediates in the adrenal steroidogenesis pathway to glucocorticoids and the sex steroids pathway to androgens and estrogens.
3. Corticosterone was detected in the majority of feathers of each type. We also detected cortisone in back feathers, androstenedione in breast feathers, and testosterone in primary feathers. Captive fledglings raised on a restricted diet had higher concentrations of corticosterone in all four feather types than captive fledglings raised on a control diet. Corticosterone concentrations were reliably repeatable across feather types when standardized for feather mass, but not for feather length. Of the seven hormones looked for in uropygial gland secretions, only corticosterone was detected in one out of 23 samples.
4. We conclude that corticosterone is the best feather‐steroid biomarker for detection of developmental nutritional stress, as it was the only hormone to manifest a signal of nutritional stress, and that exposure to stress can be compared among different feather types when corticosterone concentrations are standardized by feather mass.

## Graphical abstract

We used liquid‐chromatography tandem mass spectrometry to measure the concentration of 11 steroid hormones in four feathers grown at overlapping periods in captive reared rhinoceros auklets to determine which hormone(s) are most effective at indicating nutritional stress and whether and how hormone concentrations can be compared across feathers of different size and structure. Birds were raised on a control or restricted diet. We found that of all the hormones, corticosterone was the most reliable biomarker of exposure to nutritional stress and that when standardized for mass, concentrations of corticosterone were comparable across feathers of different sizes and structure.

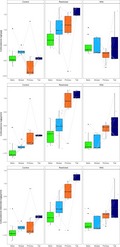

## 1 INTRODUCTION

The desire to assess the endocrine state of an animal with minimally invasive techniques has led to methods to quantify hormone concentrations in easily sampled tissues such as whiskers (Karpovich, Skinner, Kapronczai, Smith, & Janz, 2018), hair (Koren et al., 2019), and feathers (Bortolotti, Marchant, Blas, & German, 2008). In avian biology the technique of measuring stress hormones, glucocorticoids, in feathers is of particular interest as many species grow different groups of feathers at different times of the year. This feature of feathers makes them potentially valuable as indicators of physiological status during life stages when birds are inaccessible to researchers (e.g., Orben et al., 2018; Ramos, Llabrés, Monclús, López‐Béjar, & González‐Solís, 2018), over long periods of time (e.g., Fairhurst, Bond, Hobson, & Ronconi, 2015; Will, Kitaiskaia, & Kitaysky, 2018), or to assess the response of an individual to conditions over time (e.g., Ganz, Jenny, Kraemer, Jenni, & Jenni‐Eiermann, 2018; Will et al., 2015).

Bortolotti et al. (2008) posed the passive deposition hypothesis to explain how circulating glucocorticoids are incorporated into feather tissues via diffusion during keratinization and pigmentation of the feather in the blood quill (Jenni‐Eiermann, Helfenstein, Vallat, Glauser, & Jenni, 2015). Hormones may also be incorporated into feathers during preening when uropygial gland secretions, which may contain dissolved steroids, are exogenously applied to feathers and thus may accumulate as feathers age (Jenni‐Eiermann et al., 2015). Here, we test whether, as predicted by the passive deposition hypothesis, other circulating hormones and/or their metabolites are incorporated into feathers evenly throughout a bird's body and whether any other hormone is a potential candidate for measuring exposure to stress. We also address the issue of how to compare hormone concentrations across feather types, an issue regardless of how hormones arrived in the feather, and test the possibility that hormones may be incorporated into the feather via dissolved secretions in preen oils.

Corticosterone is not the only hormone transported by the circulatory system, and it is possible that other steroid hormones, including androgens and estrogens, may also be present in feather tissues. The specific steroid, and the amount, that is incorporated into feather tissues should depend on which hormones, and how much, are in circulation at the time of feather growth and may vary depending on the feather type as a result of molt schedules occurring during different life stages, take place in geographically distinct places due to migration or because a species inhabits seasonally fluctuating environments (e.g., Romero, Soma, & Wingfield, 1998). Androgen precursor steroid hormones can also be present in the circulatory system during periods of the year when synthesis in the gonads is inactive (Soma, Scotti, Newman, Charlier, & Demas, 2008). Identifying patterns in hormone deposition across feather types may, therefore, be of use to researchers hoping to target feather sampling to capture a specific seasonal representation of endocrine activity.

To date, the analysis of hormone concentrations in feathers has been largely confined to measuring the major avian glucocorticoid, and corticosterone. One previous study linked concentrations of cortisol and testosterone, in addition to corticosterone, in house sparrow flank feathers to subsequent survival (Koren et al., 2012) and another explored the detectability of testosterone (in addition to corticosterone) in very small quantities of feathers (Bílková, Adámková, Albrecht, & Šimek, 2019). However, it is largely unknown whether other steroid hormones might also be present in feather tissues and, if so, also affected by exposure to stress. “Stress” refers to a wide range of environmental conditions such as problematic temperature changes (e.g., Xie, Romero, Htut, & McWhorter, 2017), changes in food availability (e.g., Kitaysky, Wingfield, & Piatt, 1999), predation pressure (mixed evidence: Clinchy, Zanette, Boonstra, Wingfield, & Smith, 2004; Fontaine, Arriero, Schwabl, & Martin, 2011), or pollution (contaminants: Bourgeon et al., 2012; noise: Kleist, Guralnick, Cruz, Lowry, & Francis, 2018) that act directly, or indirectly via psychological stress (e.g., Cyr, Earle, Tam, & Romero, 2007), on an individual's physiology. Here, we narrowly define “stress” as developmental food limitation and build on previous work, which linked experimental exposure to nutritional stress in captive‐reared rhinoceros auklet (*Cerhorhinca monocerata*) chicks to elevated concentrations of corticosterone in plasma and feathers (Sears & Hatch, 2008; Will et al., 2014).

Whether corticosterone is the most effective biomarker to analyze in order to quantify stress exposure has received little attention (Koren et al., 2012). For example, plasma testosterone decreases in breeding male rufous‐sided sparrows *Peucaea carpalis* in response to physical restraint (Deviche et al., 2014) and plasma estrone decreases in reproductively active female black birds *Turdus merula* in response to artificial lights at night (Russ et al., 2015). In both cases, decreased concentrations of testosterone and estrone corresponded to an increase in plasma corticosterone. However, corticosterone production may be dampened during feather growth (Romero, Strochlic, & Wingfield, 2005), so in some species it may be preferable to use an alternative steroid hormone to detect exposure to environmental stress. Furthermore, the pools of precursor steroids and the metabolites produced during steroid clearance may change in response to stressful conditions.

**Figure 1:**
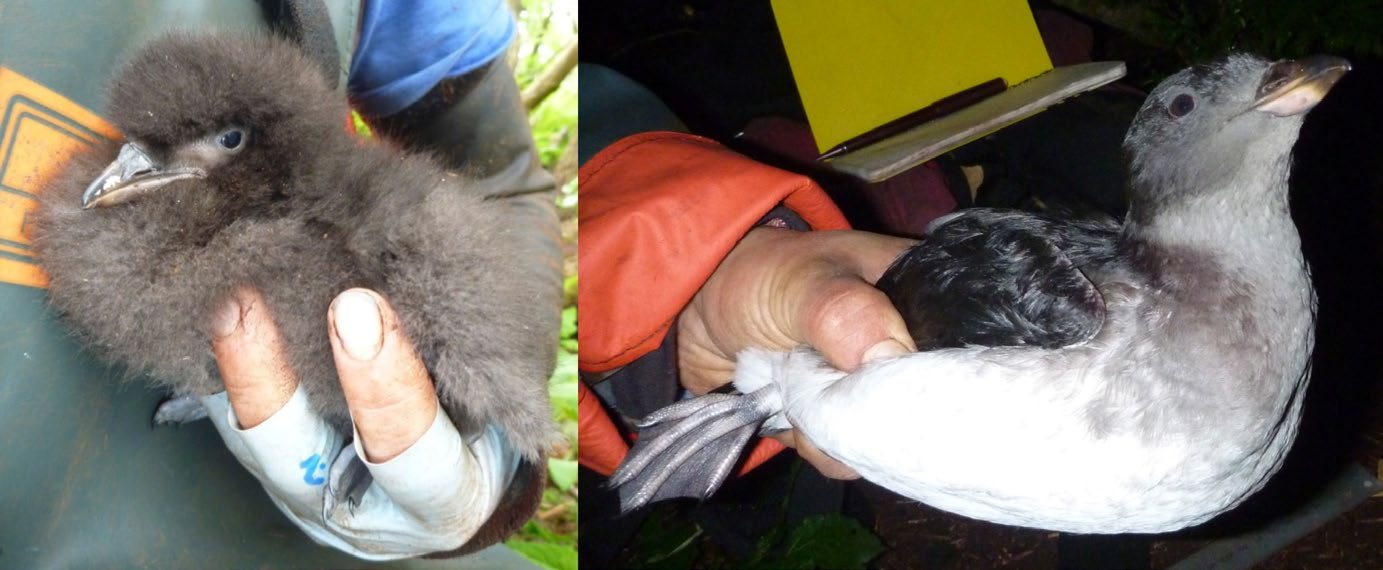
Rhinoceros auklets grow their feathers over the course of about 40 days while in the nest (photo credit: A. Will)

**Figure 2:**
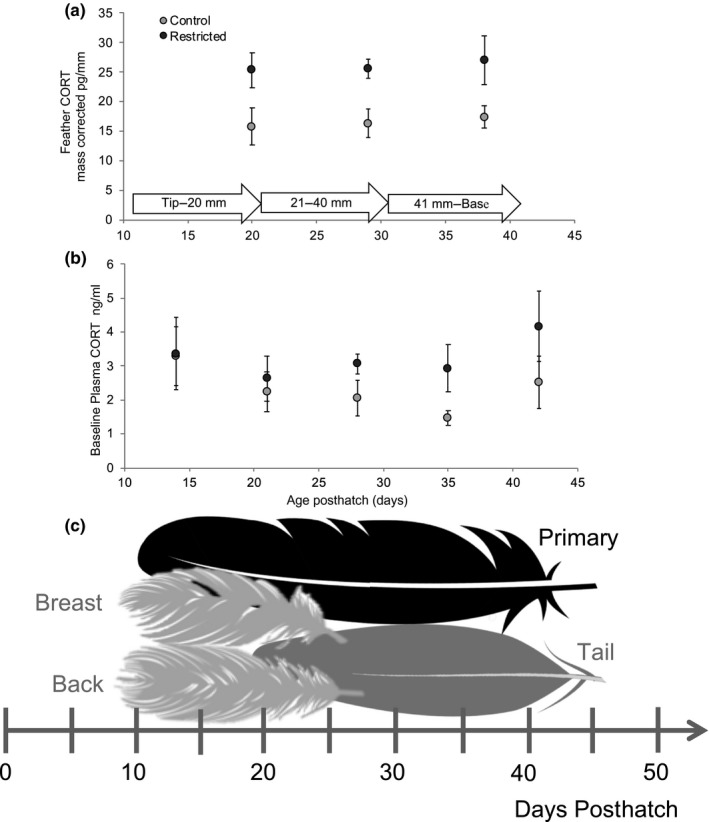
(a and b) are reproduced from Will et al. (2014), illustrating the lack of temporal patterns in the presence of corticosterone in both plasma (a) and primary feathers (b) in birds raised on a control diet (light gray) throughout the nestling period. (c) Schematic of feather growth patterns during rhinoceros auklet nestling development. Back and breast feathers emerge between day 8 and 11 and finish growing at day 24 to 28 posthatch. Primary feathers emerge at day 11 and complete growth between days 42 and 49. Tail feathers emerge at day 20 and finish growing sometime after fledging (after day 56), but for captive raised birds tail feather growth was terminated between days 42 and 48 (as illustrated above). Feathers are grown from tip (earliest) to base (latest)

Using liquid chromatography coupled to tandem mass spectrometry (LC‐MS/MS), we measured the concentrations of 11 major steroids to examine which metabolic pathways in four types of feathers (primary, tail, back, and breast), from captive rhinoceros auklet fledglings (Figure 1), grown on either food restricted or control diets. Our previous work on these same individuals showed that rhinoceros auklets experience no ontogenetic patterns in the circulation of corticosterone in plasma, or temporal trends in the concentration of corticosterone in feathers (Figure 2, Will et al., 2014). In free‐living individuals raised by their parents, temporal patterns of corticosterone deposition varied both by colony and by year and were associated with diet composition rather than age of the bird (Will et al., 2015). Due to limitations in detectability of our LC‐MS/MS method, optimized for a suite of steroids, we needed to use whole feathers, and sometimes combined several feathers in the same sample, to have sufficient concentrations. This profiles more closely the situation researchers may face in comparing whole feathers grown at different time periods in free‐living adult birds, but it may arguably sacrifice the precision that a comparison of feather segments grown at the exact same time period would yield. Since we know that there was no significant temporal ontogenetic variability in corticosterone secretion during the development of the captive‐reared chicks, we have based our whole‐feather comparison on the premise that all feathers were exposed to a relatively constant amount of circulating corticosterone in birds raised on a control diet.

Feather growth in rhinoceros auklets spans their entire nestling phase; primary feathers emerge at day 11 (Will et al., 2014) and tail feathers emerge at day 20 (A. Kitaysky, unpublished data) posthatch. In closely related tufted puffins *Fratercula cirrhata*, back and breast feathers emerge at day 8–11 posthatch (Piatt & Kitaysky, 2002). We estimate that back and breast feathers finish growing at between day 24 and 28 posthatch based on a growth rate of 2.22 mm/day, the rate of primary feather growth in captive‐reared rhinoceros auklets (Will et al., 2014). Primary feathers complete growth between day 42 and 49 and tail feathers finish growing after day 56 posthatch (A. Kitaysky, unpublished data). Captive‐reared birds were sacrificed between ages 42 and 48 when they began to regularly leave the nest (Sears & Hatch, 2008). Since all feathers overlapped in their timing of growth (Figure 2), we were able to examine whether an endocrine signal of nutritional stress was detectable across all feather types. We then used these results, with the addition of feathers collected from wild‐caught juveniles, to resolve the issue of comparing hormone concentrations across different feather types.

Bortolotti et al. (2008) originally recommended to report feather corticosterone concentrations standardized by sample length to reflect exposure to stress over time, assuming a constant growth rate, and cautioned against comparing different feather types, which may have more material, and thus more hormone, per unit of feather length. Feathers grown at different times of the year vary in size and structure, which may affect hormone deposition in the feather tissues (Harris, Madliger, & Love, 2016) and may preclude direct comparison of a bird's exposure to stressful conditions during various stages of its annual cycle. It may, however, be possible to account for differences in feather structure by standardizing concentrations by sample mass or to correct length concentrations by mass (e.g., Orben et al., 2018; Will et al., 2014; Will et al., 2015). Here, we test three methods of reporting hormone concentrations (standardized for sample length, mass, and length corrected for mass) in feathers to determine which is the least affected by the differences in feather structure among breast, back, primary, and tail feathers. Since we expect corticosterone concentrations to be similar among the four feather types, we expect that the method(s) of standardization that removes the effect of feather type on steroid concentration will show no discernable trend in steroid concentration with increasing feather size and weight.

Finally, we address the possibility that preening might deposit steroids dissolved in preening oils, including glucocorticoids, into, as well as adherent to, feathers (Jenni‐Eiermann et al., 2015). We compared steroid signatures of feathers and secretion of uropygial glands between captive fledglings which had not started preening their feathers (A. Kitaysky, personal observation, birds were sacrificed prior to prefledging preening) compared to wild juveniles (caught at sea) which have engaged in preening behavior to water‐proof their plumage additional time while at sea.

## 2 METHODS

### 2.1 Samples

We collected six back, six breast, one tail, and one primary feather by plucking, as well as uropygial gland oil by absorbent filter paper, from carcasses of captive‐reared birds of fledging age and wild‐caught juvenile rhinoceros auklets (likely a month post fledging). Captive chicks were hatched and raised at the University of Alaska Fairbanks under IACUC #06‐21 on either a control (*n* = 8) or restricted (*n* = 7) diet. Diet restriction started at day 10 posthatch and ended when chicks fledged (for experiment details see Sears & Hatch, 2008). Previous studies on these same birds reported elevated concentrations of corticosterone (based on corticosterone radioimmunoassay analyses) in both plasma (Sears & Hatch, 2008) and feathers (Will et al., 2014) of individuals raised on a restricted diet. Individuals included in this study were euthanized after fledging. Wild‐caught juveniles (*n* = 8) were collected (a by‐catch of commercial gill‐net fisheries) within a month of leaving their nests by the National Marine Fisheries Service in Southeast Alaska. All birds were stored frozen until sampled.

### 2.2 Candidate steroids quantified

**Figure 3:**
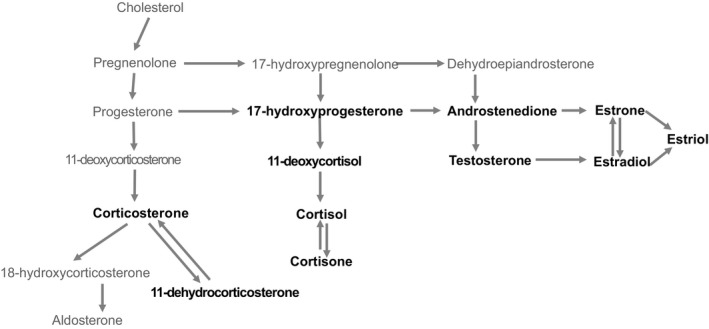
Steroidogenesis pathway adapted from Hanukoglu (2016) and Taves et al. (2011). Hormones quantified are in bold, arrows indicate direction of synthesis from substrate to product

The 11 steroids quantified encompass the pathway for adrenal glucocorticoid synthesis, as well as the sex steroid pathway to androgens and estrogens (Figure 3). Specifically, the earliest common substrate steroid for the steroidogenic pathways investigated was 17‐hydroxyprogesterone. The intermediate steroid on the adrenal steroidogenesis pathway from 17‐hydroxyprogesterone to cortisol is 11‐deoxycortisol. Finally, the first breakdown product of cortisol is cortisone, which can also serve as a substrate pool for back‐synthesis of cortisol, was quantified. In the parallel corticosteroid pathway, we were not able to quantify the direct precursors of corticosterone (progesterone and 11‐deoxycorticosterone), but were able to quantify 11‐dehydrocorticosterone as the breakdown product/substrate pool. In these parallel pathways for biosynthesis of cortisol and corticosterone, the same enzymes act on both substrates to synthesize both products (pathways described in Taves, Gomez‐Sanchez, & Soma, 2011).

17‐hydroxyprogesterone is also the immediate precursor for the gonadal sex steroid pathway. 17‐hydroxyprogesterone is converted to the androgen, androstenedione, that is, in turn, the substrate for testosterone synthesis. Estrogens are synthesized from these two androgens by the aromatase enzyme, with androstenedione as the substrate for estrone and testosterone as the substrate for estradiol. Both estrone and estradiol can then be converted into the third estrogen quantified, estriol (pathways described in Hanukoglu, 1992).

### 2.3 Chemicals and reagents

Cortisol, cortisone, corticosterone, 11‐deoxycortisol, 11‐dehydrocorticosterone, androstenedione, testosterone, 17α‐hydroxyprogesterone, estrone, estradiol, and estriol were purchased from Steroids Inc. Deuterium labeled internal standards: cortisol‐d4, corticosterone‐d8, testosterone‐d2, 17α‐hydroxyprogesterone‐d8, Estrone‐2,4,16,16‐d4, 17β‐estradiol‐2,4,16,16‐d4 (estradiol‐d4), and 16α‐Hydroxy‐17β‐estradiol‐2,4‐d2 (estriol‐d2) were obtained from CDN Isotopes Inc. Cortisone was quantified against cortisol‐d4, 11‐dehydrocorticosterone was quantified against corticosterone‐d8, and androstenedione was quantified against testosterone‐d2 because deuterated standards were not available. In each case where the calibration was against a nonbioidentical steroid, results were compared to direct reading from the nondeuterated, linear, calibration curve for the bioidentical standard and results were similar. Quantitation interference in this matrix and method precluded quantitation of progesterone. HPLC grade methanol (MeOH), Optima grade acetonitrile (ACN), ammonium fluoride (NH_4_F), and Optima grade water were purchased from Fisher Scientific.

### 2.4 Sample preparation

The calamus of each feather was removed to avoid blood and skin tissue contamination; feathers and uropygial gland secretions were stored dry at room temperature until analysis. Each feather (primary mean mass 0.036 g ± 0.004 standard deviation, tail 0.0166 g ± 0.002), group of 6 feathers (back total mean mass 0.017 g ± 0.004, breast 0.017 g ± 0.002), or uropygial gland secretion was placed in a 13 × 100 mm borosilicate test tube. To the test tube, 100 μl of combined internal standard containing all deuterated standards, plus 9 ml cold (4°C and held in an ice bath) methanol was added. Tubes containing the standards and the feathers were capped and stored in a 4°C fridge for 20 hr, whereas the filter paper samples that were explicitly oils, esters, and waxes were incubated at −20°C for 20 hr. Cold extraction reduces interfering background by reducing the extraction of nonsteroid components of the feather matrix (Di Francesco et al., 2017). After the sample was gently removed, the extract was evaporated to dryness under N_2_ at 40°C in a Techne Sample Concentrator and reconstituted with 150 (back and breast feathers of all birds, and primaries of captive‐reared birds) or 200 (tail feathers of all birds and primaries of wild‐caught birds) µl of H_2_O/MeOH (50/50, v/v). The sample was centrifuged at 18,000 × *g* (Legend micro‐21R, Thermo Scientific) for 15 min, and 100 or 150 µl of its supernatant was submitted for LC‐MS/MS.

### 2.5 LC‐ESI/MRM method

All samples were analyzed using an Agilent 1200 binary liquid chromatography (LC) system connected with an AB SCIEX QTRAP^®^ 5500 tandem mass spectrometer, equipped with an electrospray ionization (ESI) source. LC separation was performed on an Agilent ZORBAX Eclipse plus C18 column (100 × 2.1 mm, 1.8 µm particle size) at 40°C. Mobile phase A was ACN/H_2_O (5/95, v/v, 2 mM NH_4_F), and the mobile phase B was 100% ACN (2 mM NH_4_F). The 12 min gradient for Positive ESI mode was 15%–70% B (0–6 min), 70%–100% B (6–7 min), 100% B (7–8.5 min), and 100%–15% B (8.5–9 min), held at 15% B for 3 min. The 9 min gradient for Negative ESI mode was 40%–80% B (0–4 min), 80%–100% B (4–4.5 min), and 100% B (4.5–5.5 min), 100%–40% B (5.5–6 min), held at 40% B for 3 min. The flow rate was 0.22 ml/min, and the injection volume was 12 µl.

Ionization conditions for ESI + were as follows: Curtain gas, 45 psi; Temperature, 600°C; Ion Source Gas 1, 40 psi; Ion Source Gas 2, 30 psi; Collision Gas, Medium; Ionspray Voltage, 5,000 V. Ionization conditions for ESI− were as follows: Curtain gas, 35 psi; Temperature, 700°C; Ion Source Gas 1, 40 psi; Ion Source Gas 2, 55 psi; Collision Gas, Medium; Ionspray Voltage, −4,500 V.

Mass resolutions in Q1 and Q3 were set to unit resolution. Each analyte was monitored by two transitions (a quantifier and a qualifier). For ESI +: Cortisol 363/121, 363/327; Cortisol d‐4 367/121, 367/331; Corticosterone 347/329, 347/121; Corticosterone d‐8 355/337, 355/125; Cortisone 363/163, 361/121; 11‐dehydrocorticosterone 345/121, 354/301; Testosterone 289/91, 289/109; Testosterone‐d2 291/99, 291/111; 17‐hydroxyprogesterone 331/97, 331/109; 17‐hydroxyprogesterone‐d8 339/100, 339/109; 11‐deoxycortisol 347/97, 347/109; Androstenedione 287/97, 287/109. For ESI−: estrone 269/145, 269/143, estrone‐d2 273/147, 273/145; estradiol 271/145, 271/183; estradiol‐d4 275/147, 275/145; estriol 287/171, 287/145; estriol‐d2 289/173, 289/147. Declustering potential was 100V for ESI+ and −120 V for ESI−. Entrance potential was 3 V for ESI+ and −10 V for ESI−. Collision energies ranged from 24 to 32 eV for ESI+ and were −53 V for ESI−. Collision cell exit potential was 12 V for ESI+ and −18 V for ESI−.

### 2.6 Quantitation method

Each run contained a 10‐point calibration series for each steroid. The range was from 100 ng/ml down to 0.1 ng/ml for cortisol, corticosterone, cortisone, and androstenedione and 50 ng/ml down to 0.05 ng/ml for the remaining steroids. The lowest calibration standard that gave <20% CV and <30% error was the lower limit of quantitation (LLOQ). LLOQ was 0.1 ng/ml for all steroids except cortisol and corticosterone, with an LLOQ of 0.2 ng/ml. When the sample fell below LLOQ, no concentration was reported. Three quality control solutions at 5.0/0.5/0.1 ng/ml or 2.5/0.25/0.05 ng/ml were included in each run. All CVs for all QC above LLOQ were <20%.

Each steroid was quantified relative to an internal standard that carried deuterium substitutions for hydrogen that increased the molecular weight without altering the biochemical properties of the molecule. Calibration curves used analyte/internal standard peak area ratios (*y* axis) versus analyte concentration (*x* axis) yielding a linear fit with 1/*x* weighting to improve precision at low concentrations. *R* ^2^ for all calibration lines were always higher than 0.995, and typically higher than 0.998.

### 2.7 Standardization of concentrations

We standardized hormone concentrations (Table 1) by mass ([ng/ml]*reconstituted volume/sample mass) and length ([ng/ml]*reconstituted volume/ sample length) and length detrended for mass. Standardized concentrations were log transformed to meet assumptions of normality. To remove the effect of mass on the standardized by length concentrations, we calculated the residuals for each data point using the linear regression of log‐transformed feather masses and log‐transformed standardized by length corticosterone concentrations. This retains the variability in the data, but allows us to remove the positive linear trend associated with an increase in sample mass. We then converted the residuals back into concentrations for reporting purposes by adding each residual to the predicted corticosterone concentration for the mean sample mass calculated using the linear regression above. For the mixed effects model (description below), we used the lower level of quantitation (LLOQ) value for samples with ng/ml concentrations below the LLOQ.

**Table 1:** Raw steroid concentrations including minimum (or LLOQ) and maximum by feather type

	Cortisol (ng/ml)	Cortisone (ng/ml)	11‐DHC (ng/ml)	Corticosterone (ng/ml)	Testosterone (ng/ml)	17‐OH‐Progesterone (ng/ml)	Androstenedione (ng/ml)	Estradiol (ng/ml)
Back								
Mean	0.215	0.099	0.052	0.658	0.089	LLOQ	0.345	LLOQ
Range	<0.2–0.427	<0.1–0.365	<0.1–0.103	<0.2–2.6915	<0.1–0.215	<0.1	<0.1–1.71	<0.1
Breast								
Mean	0.547	0.158	0.176	1.375	0.159	LLOQ	0.295	LLOQ
Range	<0.2–1.42	<0.05–0.24	<0.1–0.247	<0.1–4.45	<0.05–0.283	<0.1	<0.1–1.66	<0.1
Primary								
Mean	LLOQ	0.052	LLOQ	2.400	0.196	0.063	0.158	0.087
Range	<0.2	<0.05–0.0523	<0.1	<0.2–9.222	<0.1–0.801	<0.1–0.0794	<0.1–0.664	<0.1–0.1297
Tail								
Mean	0.821	0.512	LLOQ	2.394	0.311	LLOQ	0.260	LLOQ
Range	<0.2–1.58	<0.05–0.512	<0.1	<0.1–4.56	<0.05–0.973	<0.1	<0.1–0.517	<0.1

Note

17‐OH‐Progesterone is the same as 17‐hydroxyprogesterone. 11‐DHC is the abbreviation for 11‐dehydrocorticosterone. Estriol and Estrone were not detected in any sample.

### 2.8 Statistical analysis

All analyses were performed in R v. 3.4.2 (R Core Team, 2017). We used linear mixed effects models to evaluate which method of standardization, by length, mass, or detrending length concentrations for mass, results in patterns of corticosterone concentrations of back, breast, primary, and tail feathers most similar to previously observed patterns in plasma corticosterone concentrations, where nestlings during primary feather growth do not have higher concentrations of circulating corticosterone than during body feather growth. These three standardized concentrations were modeled with linear mixed effects models using the *lme4* (Bates, Maechler, Bolker, & Walker, 2015) and *lme4Test* (Kuznetsova, Brockhoff, & Christensen, 2017) packages in R. We included feather type (“FeatherType” = back, breast, primary) as a fixed effect and individual as a random intercept.

## 3 RESULTS

### 3.1 Steroid detection by feather type

The common cortisol and sex steroid precursor 17‐hydroxyprogesterone were detected in fewer than half of the feather samples, irrespective of sex (*n* = 23 individuals for all analyses except for primary feathers for estradiol and estrone *n* = 15, and tail feathers were not analyzed for estradiol and estrone, see Table 2 for detection summary). The product of 17‐hydroxyprogesterone, 11‐deoxycortisol, was not detected in any of the feathers analyzed. Cortisol, the product of 11‐deoxycortisol, was detected in only a few back (4.35%) and breast (13.04%) feathers. Cortisone, a product of cortisol, was detected in a higher proportion of back feathers (73.91%) than in breast (17.39%), tail (4.35%), and primary (4.35%) feathers (Fisher's exact test: *p* < .001). In the parallel corticosteroid pathway, corticosterone was detected in half or more of the feathers of each type. Its product 11‐dehydrocorticosterone, which can also serve as a substrate pool, was detected in less than half of the feathers of each type. Androstenedione, the sex steroid precursor, was detected in breast (73.91%), back (34.78%), and primary (52.17%) feathers but only in a limited number of tail feathers (17.39%, Fisher's exact test: *p* < .001) while testosterone, one of its products, was detected in nearly all primary feather samples (91.30%), but in fewer than half of all other feather types analyzed (Fisher's exact test: *p* < .001). Estradiol, a product of testosterone, was detected in fewer than half of the primary feathers and was not detected in body feathers. The other estrogens (estrone and estriol) were not detected.

**Table 2:**
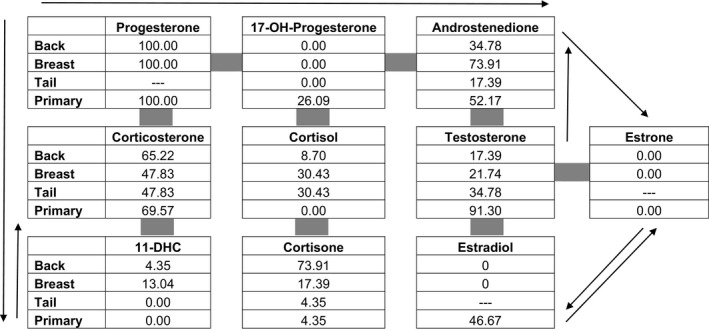
The percentage of samples in which each hormone was detected above the lower limit of quantitation

Note

Estradiol was detected in a higher proportion of female feathers than male feathers. Estriol, not depicted, was not detected in back, breast, or primary feathers. Tail feathers were not analyzed for estrone, estradiol, or estriol. 17‐OH‐Progesterone is the same as 17‐hydroxyprogesterone. 11‐DHC is the abbreviation for 11‐dehydrocorticosterone.

### 3.2 Effect of sex

**Figure 4:**
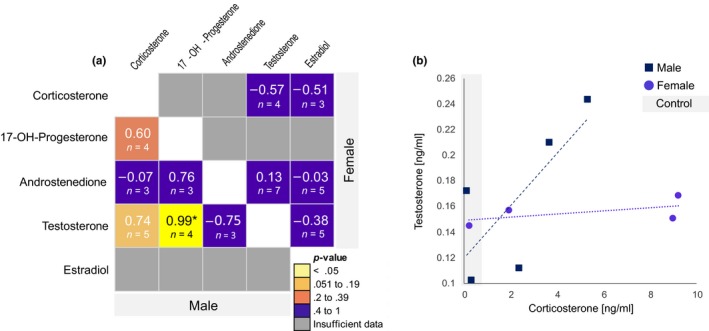
Association of raw steroid hormone concentrations measured in primary feathers of male and female rhinoceros auklet captive‐reared fledglings. (a) Pearson correlation coefficient matrix for male (lower left) and female (upper right) fledglings. Sample sizes are noted below coefficients and boxes are color‐coded according to linear regression p‐values. “*” indicates that the relationship remains significant when male and female concentrations are combined. (b) The linear regression associations between raw corticosterone and testosterone concentrations in male (dark blue squares) and females (purple circles). Shaded gray box indicates data points from birds on a control diet, and sample sizes were too small to include treatment as a factor in steroid concentration comparisons

The proportion of samples in which steroids were detected did not significantly differ between male and female birds (captive‐reared and wild‐caught combined). Although not statistically significant, proportions of detection did vary. For example, 17‐hydroxyprogesterone was detected in more male (50% *n* = 8) than female birds (20%, *n* = 12, Fisher's exact test: *p* = .16) and estradiol was detected in more female (50%, *n* = 12) than male birds (12.5%, *n* = 8, Fisher's exact test: *p* = .15). Testosterone was detected in all but one bird, a female, and androstenedione was detected in half of both female and male birds (58%, *n* = 12 and 50%, *n* = 8 respectively, Fisher's exact test: *p* = 1 for both steroids). To determine whether hormones may be acting in tandem or serving as a potential substrate pool, we examined the correlation matrix of detected hormones (Figure 4, Wynne‐Edwards, Lee, Zhou, & Edwards, 2019). In primary feathers, there was a positive association between the concentration of testosterone and androstenedione (both sexes *n* = 6, Pearson's correlation coefficient *r* = .99, simple linear regression *F*_1,4_ = 340.8, *p* < .001). Corticosterone and testosterone concentrations were positively correlated in male (*r* = 0.74, *F*_1,3_ = 3.68, *p* = .15) but less so in female primary feathers (*r* = .57, *F*_1,2_ = 0.95, *p* = .43, Figure 4).

### 3.3 Measuring exposure to nutritional stress

Back, breast, primary, and tail feathers from birds raised on a restricted diet (*n* = 7) had significantly higher concentrations of corticosterone than control birds (*n* = 8, Wilcoxon sign rank test: *W* > 6, *p* < .035, Table 3). No other hormone had concentrations that significantly differed between the two treatments.

**Table 3:**
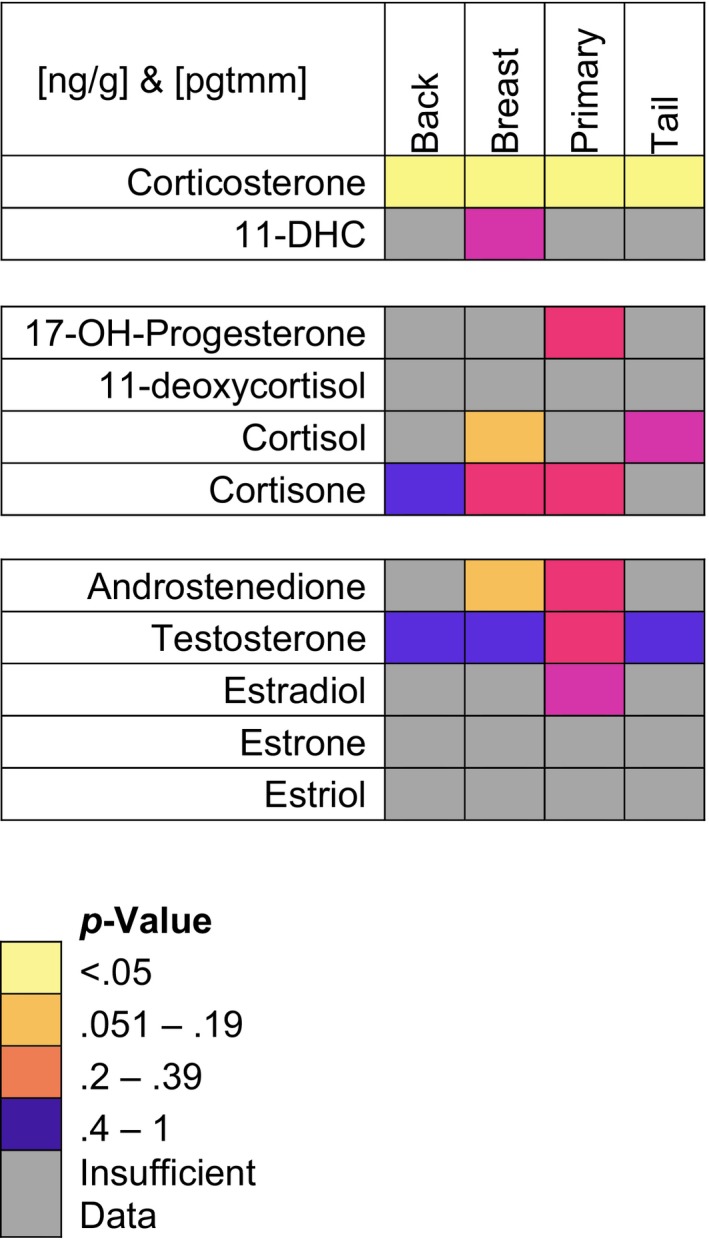
Matrix of *p*‐value results of Wilcoxon sign rank tests comparing hormone concentrations in feathers grown by rhinoceros auklet chicks raised on a control compared to a restricted diet

Note

Comparisons between treatments were only made within a given feather type. Gray indicates that there were insufficient data to make a comparison. Colors indicate the range of the *p*‐value. Warmer colors indicate lower *p*‐values and a greater difference in concentrations between the two treatment groups. All *p*‐values in the 0.051 to 0.19 bracket were >0.13. *p*‐Value color‐assignment is the same for concentrations standardized by feather mass and feather length, and results for both are represented in this table. 11‐DHC is the abbreviation for 11‐dehydrocorticosterone; 17‐OH‐Progesterone is an abbreviation for 11‐hydroxyprogestererone.

**Figure 5:**
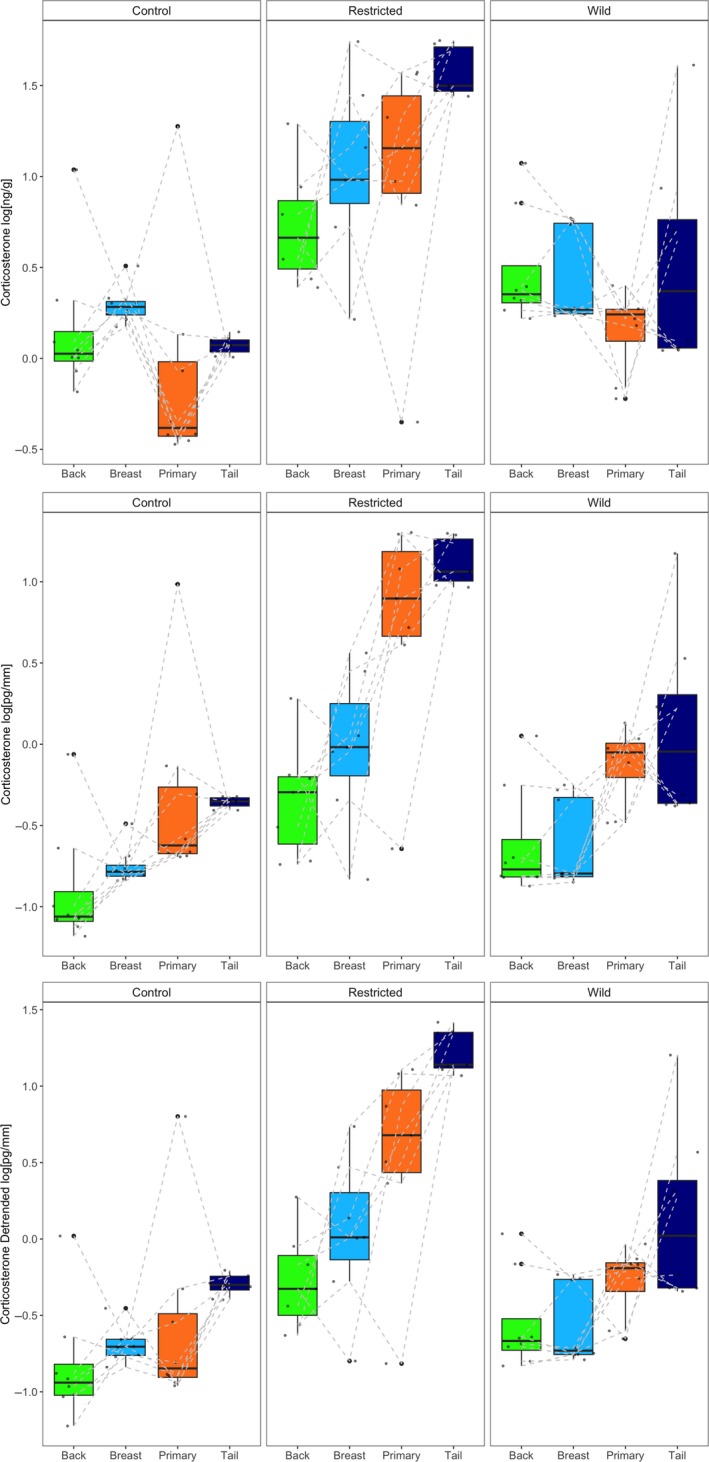
Boxplots depict the median and distribution of corticosterone concentrations standardized by sample weight (top), length (middle), and length detrended for mass (bottom) of back, breast, primary, and tail feather samples from captive and wild reared (*n* = 8) rhinoceros auklets. Birds raised in captivity were fed either a control (*n* = 8) or restricted (*n* = 7) diet. Raw data are overlaid, and individuals are connected with a light gray dashed line

Birds raised on a restricted diet had higher feather corticosterone concentrations than control birds across all methods of standardization (“Treatment” by mass: *F*_2,20_ = 15.73, *p* < .0001, length: *F*_2,20_ = 15.95, *p* < .0001, and length detrended for mass: *F*_2,20_ = 16.03, *p* < .0001; Figure 5). There was a greater difference in feather corticosterone concentrations in primary and tail feathers in food‐restricted birds than in control and wild birds across all three methods of standardizing concentrations (“Treatment*FeatherType” by mass: *F*_6,60_ = 3.105, *p* = .01, length: *F*_6,60_ = 2.90, *p* < .001, length detrended for mass: *F*_6,60_ = 3.024, *p* = .012, Figure 5). Feather type was not a significant variable when concentrations were standardized for mass (*F*_2,40_ = 2.55, *p* = .09) but was when concentrations were standardized for length (*F*_2,40_ = 24.83, *p* < .0001) and detrended for mass (*F*_2,40_ = 9.44, *p* = .0004). If a standardization method removes the effect of feather type, we would expect to see no systemic increase in steroid concentrations as feather size increases. Concentrations of corticosterone in primary and tail feathers were no different (*p* > .05) than concentrations in breast and back feathers when concentrations were standardized for mass. When concentrations were standardized by length detrended for mass, only the tail feathers differed from back feather corticosterone concentrations (*p* < .01, Figure 5, see Table 4 for effect sizes and *t* tests).

**Table 4:** Effect sizes and standard errors estimated by linear mixed effect models of feather corticosterone concentrations standardized by mass (log [ng/g]), length (log [pg/mm]) and length detrended for mass (detrended log [pg/mm])

	log [ng/g]				log [pg/mm]				Detrended log [pg/mm]			
	Estimate	±*SE*	*t*	*p*	Estimate	±*SE*	*t*	*p*	Estimate	±*SE*	*t*	*p*
*Intercept*	0.156	0.14	1.09	.28	**−0.901**	**0.14**	**−6.28**	**<.01**	**−0.832**	**0.14**	**−5.82**	**<.01**
Restricted	**0.567**	**0.20**	**2.72**	**.01**	**0.560**	**0.20**	**2.67**	**<.01**	**0.561**	**0.20**	**2.68**	**<.01**
Wild	0.323	0.19	1.60	.11	0.282	0.20	1.39	**.17**	0.277	0.19	1.37	.18
Breast	0.137	0.18	0.78	.44	0.152	0.18	0.84	.40	0.139	0.18	0.78	.44
Primary	−0.252	0.18	−1.43	.16	**0.557**	**0.18**	**3.09**	**<.01**	0.263	0.18	1.47	.15
Tail	−0.086	0.18	−0.49	.63	**0.542**	**0.18**	**3.06**	**<.01**	**0.532**	**0.18**	**2.99**	**<.01**
Restricted:Breast	0.175	0.26	0.68	.50	0.163	0.26	0.62	.54	0.172	0.26	0.66	.51
Wild:Breast	−0.175	0.25	−0.70	.49	−0.153	0.25	−0.60	.55	−0.149	0.25	−5.90	.56
Restricted:Primary	**0.541**	**0.26**	**2.10**	**.04**	**0.535**	**0.26**	**2.03**	**.05**	**0.550**	**0.26**	**2.10**	**.04**
Wild:Primary	−0.074	0.25	−0.30	.77	−0.065	0.25	−0.25	.80	0.015	0.25	0.06	.95
Restricted:Tail	**0.942**	**0.26**	3.65	<.01	**0.921**	**0.26**	**3.55**	**<.01**	**0.963**	**0.26**	**3.69**	**<.01**
Wild:Tail	0.125	0.25	0.50	.62	0.167	0.25	0.67	.51	0.166	0.25	0.66	.52

Note

Comparisons are made to the control treatment and back feathers, *t*‐values and associated probabilities (*p*‐values) were estimated using the *lmerTest* package to facilitate interpretation of results, *p*‐values less than .05 are highlighted in bold.

### 3.4 Detection of steroids in preen oil

Corticosterone was detected in only one uropygial oil sample from a food restricted captive bird, although this individual did not have the highest feather corticosterone concentrations. There was no detection of 17‐hydroxyprogesterone, 11‐deoxycortisol, cortisol, cortisone, 11‐dehydrocorticosterone, androstenedione, or testosterone in any of the preen oil samples (*n* = 23).

## 4 DISCUSSION

We used LC‐MS/MS to quantify the concentrations of 11 steroid hormones in different feather groups grown by captive‐reared fledglings and wild‐caught juvenile rhinoceros auklets to test four predictions of the passive deposition hypothesis regarding the presence, signal of nutritional stress, and comparability of steroid hormones in and across feather types, as well as the possibility that steroid hormones may also be incorporated into feathers exogenously via preen oils.

We sampled feather types grown at overlapping intervals as a method of assessing the size of the steroid pool in a feather. We found that androstenedione and testosterone were detected in each of the four feather types: back, breast, primary, and tail that we tested. In the corticosteroid pathways, corticosterone and cortisone were also detected in each of the four feather types. With the exception of corticosterone, however, the proportion of detection of a particular steroid varied by feather type. Androstenedione was seldom detectable in tail feathers, testosterone was mostly detected in primary feathers, and cortisone was detected in a higher proportion of back feathers compared to the other feather types.

Assuming production of steroids is systemic, variability in the detection of hormones across feather types may be due to ontogenetic changes in the steroids in circulation. Although the four feathers we investigated overlap during their growth, primary and tail feathers begin growing slightly later and continue growing for longer than back or breast feathers (Figure 2). While this difference in timing does not explain differences in detection of cortisone in back and breast feathers, it may account for differences in the other steroids. In Greylag geese *Anser anser* the presence of androgen, glucocorticoid, and estrogen metabolites changed throughout the nestling period (Frigerio, Moestl, & Kotrschal, 2001). Glucocorticoids were present throughout development, while androgens and estrogens appeared at days 20 and 40, respectively (Frigerio et al., 2001). These patterns are reflected in the differential presence of these steroids in the rhinoceros auklet feathers (Table 2) and may be associated with developmental stress (glucocorticoids) and the development of the gonads and sexual behaviors (androstenedione, testosterone, and estradiol; Frigerio et al., 2001). Thus, the variability in the presence and the levels of hormones across feather types may be due in part to whole‐organism differences in steroid profiles during the nestling period.

It is possible that differences in detection across feather types are due to local production of steroids. In altricial zebra finches (*Taeniopygia guttata*) lymphoid organs produce cortisol (and its precursors) during early development prior to the adrenals becoming fully responsive (Taves et al., 2016) and was found to be an important hormone in the development of the immune system (Schmidt and Soma, 2008). Rhinoceros auklets are, however, semiprecocial, and similar lymphoid production of glucocorticoids was not found in precocial Japanese quail or chickens (Taves et al., 2016). It is not known whether steroids may be locally produced elsewhere, for example, in the skin or the feather follicle itself. Another possible explanation is that local metabolism of hormones, perhaps at the feather follicle (Rettenbacher, Henriksen, Groothuids, & Lepschy, 2013), changes the type and availability of hormones present in the blood that circulates in the feather pulp, where hormone incorporation into the keratin structure of the feather was shown to occur (Jenni‐Eiermann et al., 2015). Local metabolism may occur at different rates or express different steroidogenic enzymes, which may result in different end products.

Only corticosterone concentrations manifested a signal of nutritional stress, indicating that steroidogenesis is not affected by nutritional deficit. Rhinoceros auklet chicks raised on a restricted diet had higher concentrations of corticosterone than their counterparts raised on a control diet. Previous work has linked elevated corticosterone concentrations in developing birds that retain a responsive hypothalamic‐pituitary‐adrenal function to food restriction (e.g., Kitaysky, Kitaiskaia, Wingfield, & Piatt, 2001; Sears & Hatch, 2008), increased thermogenesis (e.g., López‐Jiménez et al., 2016), sibling hierarchy (e.g., López‐Jiménez et al., 2016), and interactions between prenatal hormone exposure and postnatal food restriction (e.g., Benowitz‐Fredericks, Schultner, & Kitaysky, 2015). Although we did not find another candidate hormone for reflecting continuous exposure to nutritional stress, we did find that high concentrations of testosterone were associated with high levels of corticosterone in male fledglings. In pied flycatcher (*Ficedula hypoleuca*), nestlings dosing with oral testosterone increased begging behavior (Goodship & Buchanan, 2007). It is not known whether testosterone is associated with begging behavior in rhinoceros auklet nestlings, however, it is possible that the positive association of testosterone and corticosterone concentrations in the feathers of male chicks (Figure 4) may be an adaptation of male nestlings to counteract preferential maternal investment in female offspring observed during poor environmental conditions (Addison, Kitaysky, & Hipfner, 2008).

We used the concentrations of corticosterone in feathers naturally grown at overlapping time periods in captive‐reared fledglings and wild‐juvenile rhinoceros auklets to address the lingering question of whether it is meaningful to compare hormone concentrations across feather types. Lattin, Reed, Desrochers, and Romero (2011) found that hormone concentrations were very high for very small feather masses, an artifact that has not been explained by extraction efficiency (Berk, McGettrick, Hansen, & Breuner, 2016). Harris et al. (2016) compared simultaneously grown feathers in adult wild starlings and found that feather corticosterone concentrations were not comparable when standardized for feather length, however, when Lendvai, Giraudeau, Németh, Bakó, and McGraw (2013) used the per mass standardization to compare tail and breast feather corticosterone concentrations in house finches (*Haemorhous mexicanus*) the results were consistent within a given individual. We found that, when mass is included in the standardization, concentrations of corticosterone were comparable across back, breast, primary, and tail feathers and that patterns associated with the amount of feather material were eliminated (Figure 3), but that the signal of nutritional stress in restricted birds was still evident. When standardized for mass, an increase in concentrations of corticosterone with feather mass was still apparent in the food restricted group but not in controls. Increased nutritional stress later in the nestling period in the restricted treatment may have occurred as the chicks' energy requirements grew, but the amount of food available remained the same an experimental decision made to mimic parental provisioning in the wild (Figure 2, Sears & Hatch, 2008). This signal was not detected in measures of plasma corticosterone concentrations in food restricted birds (Figure 2). Measurements of corticosterone in plasma of the captive‐reared rhinoceros auklets used in this study mirror the results obtained when concentrations are standardized for mass alone. It is critical to note that the feathers analyzed, although they overlapped in growth, did not strictly grow concurrently. Hormone concentrations standardized for length and then detrended for mass yielded similar results to comparisons made when concentrations were standardized for mass alone, but did not correct for the largest differences between light, flimsy body feathers, and dense, robust tail feathers.

We evaluated whether dissolved steroid hormones may be incorporated into the feathers from exogenously applied preening oils. We detected only corticosterone concentrations in the preen oil of a single individual out of 23 individuals tested. We did not detect any of the other focal hormones targeted in our analysis in any of the other uropygial gland secretions. Previously, corticosterone was also not detected in preen oils using an immunoassay (Lattin et al., 2011). Hormones were not detected in preen oils sampled from wild juvenile, individuals who had begun to apply preen oil to their feathers. Thus, our study supports the original conclusions by Bortolotti et al. (2008) and does not provide evidence that dissolved steroid hormones are incorporated into feather tissues (or contribute to hormones measured after extraction from feathers) via the exogenous application of preen oils.

In general, our findings are somewhat limited by the sensitivity (LLOQ) of the quantitation method and the extent of interfering compounds in the extraction supernatant. Optimization of a method for simultaneous quantitation of 11 hormones in two runs (ESI+ and ESI−) necessarily resulted in less sensitive detection for any single steroid. In addition, the high specificity of mass spectrometry often results in lower concentrations than an immunoassay, unless sample separation and purification are used to minimize the presence of potentially cross‐reacting compounds in the sample (Faupel‐Badger et al., 2010). In mass spectrometry, increasing the number of feathers extracted cannot circumvent this challenge, because background increases with the signal (Berk et al., 2016). The cold extraction methods used here were specifically chosen to reduce extraction of interfering compounds, yielding improvements in background (Di Francesco et al., 2017).

## 5 SUMMARY AND CONCLUSION

We used a highly specific technique to identify the suite of hormones present in four types of feathers in a developing seabird. We found that, even in newly fledged chicks, corticosterone was detectable in over half of the feathers analyzed and that androstenedione, testosterone, and cortisone were reliably detectable in some feather types but not others. This variability in detection of hormones across feather types followed previously reported patterns of steroid presence during nestling development. The signal of food restriction was most clearly detected in corticosterone concentrations across all feather types, and birds experiencing food restriction had higher concentrations of corticosterone. Finally, we used feathers grown during overlapping periods of development to test which method of standardizing hormone concentrations was most appropriate for comparing concentrations across different feather types. Standardizing by mass minimized the effect of feather size and type on the concentration of corticosterone.

## CONFLICT OF INTEREST

The authors have no conflicts of interest to declare.

## AUTHOR CONTRIBUTIONS

AK and KW‐E conceived this study, KW‐E and RZ conducted the analysis, and AW and AK analyzed the results. All authors contributed to the writing of the final manuscript.

## Data Availability

Data are available through the Dryad Digital Repository, https://doi.org/10.5061/dryad.83533nh.
